# Multidrug-resistant extended spectrum β-lactamase (ESBL)-producing *Escherichia coli* from farm produce and agricultural environments in Edo State, Nigeria

**DOI:** 10.1371/journal.pone.0282835

**Published:** 2023-03-10

**Authors:** Etinosa O. Igbinosa, Abeni Beshiru, Isoken H. Igbinosa, Gyu-Sung Cho, Charles M. A. P. Franz

**Affiliations:** 1 Faculty of Life Sciences, Applied Microbial Processes & Environmental Health Research Group, University of Benin, Benin City, Nigeria; 2 Department of Microbiology, College of Natural and Applied Sciences, Western Delta University, Oghara, Delta State, Nigeria; 3 Faculty of Life Sciences, Department of Environmental Management & Toxicology, University of Benin, Benin City, Nigeria; 4 Department of Microbiology and Biotechnology, Max Rubner–Institut, Federal Research Institute of Nutrition and Food, Kiel, Germany; North Carolina State University, UNITED STATES

## Abstract

Antimicrobial resistance (AMR) is a major public health concern, especially the extended-spectrum β-lactamase-producing (ESBL) *Escherichia coli* bacteria are emerging as a global human health hazard. This study characterized extended-spectrum β-lactamase *Escherichia coli* (ESBL-*E*. *coli*) isolates from farm sources and open markets in Edo State, Nigeria. A total of 254 samples were obtained in Edo State and included representatives from agricultural farms (soil, manure, irrigation water) and vegetables from open markets, which included ready-to-eat (RTE) salads and vegetables which could potentially be consumed uncooked. Samples were culturally tested for the ESBL phenotype using ESBL selective media, and isolates were further identified and characterized via polymerase chain reaction (PCR) for β-lactamase and other antibiotic resistance determinants. ESBL *E*. *coli* strains isolated from agricultural farms included 68% (17/25) from the soil, 84% (21/25) from manure and 28% (7/25) from irrigation water and 24.4% (19/78) from vegetables. ESBL *E*. *coli* were also isolated from RTE salads at 20% (12/60) and vegetables obtained from vendors and open markets at 36.6% (15/41). A total of 64 *E*. *coli* isolates were identified using PCR. Upon further characterization, 85.9% (55/64) of the isolates were resistant to ≥ 3 and ≤ 7 antimicrobial classes, which allows for characterizing these as being multidrug-resistant. The MDR isolates from this study harboured ≥1 and ≤5 AMR determinants. The MDR isolates also harboured ≥1 and ≤3 beta-lactamase genes. Findings from this study showed that fresh vegetables and salads could be contaminated with ESBL-*E*. *coli*, particularly fresh produce from farms that use untreated water for irrigation. Appropriate measures, including improving irrigation water quality and agricultural practices, need to be implemented, and global regulatory guiding principles are crucial to ensure public health and consumer safety.

## Introduction

The usage of antimicrobial drugs in veterinary and human medicine has led to the extensive development of antimicrobial resistance (AMR) in animals, humans, and the environment [1–3]. A significant concern is how AMR disseminates rapidly globally [4,5]. Third-generation cephalosporins are essential in our defence against bacterial infections [6]. Exposure to 3^rd^ generation cephalosporin-resistant *Escherichia coli* (*E*. *coli*) may occur through different routes, such as interaction with animal or human carriers, consuming contaminated food or interaction with contaminated environmental compartments [7]. It is pivotal to elucidate the relative involvement of probable routes and sources to institute preventive mechanisms.

Various market segments, from consumers to restaurants and institutional markets, are regularly supplied with fresh produce in Nigeria. An enormous quantity of ready-to-eat (RTE) vegetables is retailed, particularly street foods [8]. Several outbreaks have highlighted fresh vegetables as potential vehicles of foodborne disease [9]. Although not in outbreak proportions, *E*. *coli* illnesses have also been reported in Southern Nigeria [10–12]. Its heightened concern over the spread of antimicrobial-resistant pathogens made this research imperative. Nigeria currently does not have a functional foodborne disease reconnaissance system. Its direct healthcare consequence is projected at approximately $3 billion, responsible for 17–25% of the projected cost for all illnesses [13]. Nigeria’s Area Councils Health System profile showed foodborne infection (FBI) as the foremost cause of mortality, averaging 25%. In Nigeria, >200,000 people die annually from food poisoning caused by foodborne pathogens, as estimated by the World Health Organization (WHO) [2,8,14]. Mortalities result from contaminated foods via improper preservation, service and processing [15].

Fecal *E*. *coli* can occur in the soil, water and on plants. ESBL-producing pathotypes of *E*. *coli* have also been recovered in the environment [16–18]. Since vegetables are usually eaten raw, ingesting ESBL-producing *E*. *coli* that can reach the gastrointestinal tract poses a potential public health risk [6,14,19]. In recent decades, the prevalence of multidrug resistance (MDR) *E*. *coli* has been increasing globally [20,21]. Selective pressure from antibiotics, which culminates from their usage and abuse, contributes to AMR [3,22]. A key mechanism for resistance to third-generation cephalosporins is centred on the plasmid-mediated enzyme (β-lactamase) production, which inactivates these compounds by cleaving the β-lactam ring. CTX-M is one of the most predominantly documented ESBL β-lactamase types from clinical environments in *E*. *coli* isolates [23].

ESBL-producing *E*. *coli* from animals and humans can enter the environment through manure and sewage. Fresh produce may become contaminated with ESBL-producing *E*. *coli* from the farm environment by irrigation with contaminated water sources or when cultivated in manure-supplemented soil. ESBL-producing *E*. *coli* could persist on vegetable surfaces and sometimes advance to the interior and spread to animals or humans [24,25]. In recent years, interest has focused on the microbiological hazards that challenge the food industry [1,8,14]. It is imperative to pinpoint the crucial role of consuming uncooked or undercooked vegetables in bacterial gastroenteritis [26]. There are limited studies on spreading and transmitting ESBL-producing *E*. *coli* in agricultural environments and food vegetables [7,27]. The incidence of ESBL *E*. *coli* in food, vegetables, and the farm environment is detrimental to public health and food safety. The current study intended to evaluate the presence of ESBL-producing *E*. *coli* on food vegetable samples in Edo State, Nigeria. Ready-to-eat (RTE) salads and vegetables that are potentially consumed uncooked were selected for isolation and characterization of ESBL *E*. *coli* isolates, as were vegetables from farms and markets. Vegetables from farms were included to reflect the occurrence of ESBL-producing *E*. *coli* in the agricultural environment.

## Materials and methods

### Study area and sample collection

The sample size for this study was determined using the sample size determination formula below:

$$Sample\;(N)=\frac{\left(Z_{1-\propto /2}{)}^{2}P(1-P\right)}{d^{2}}$$

**Fig 1:**
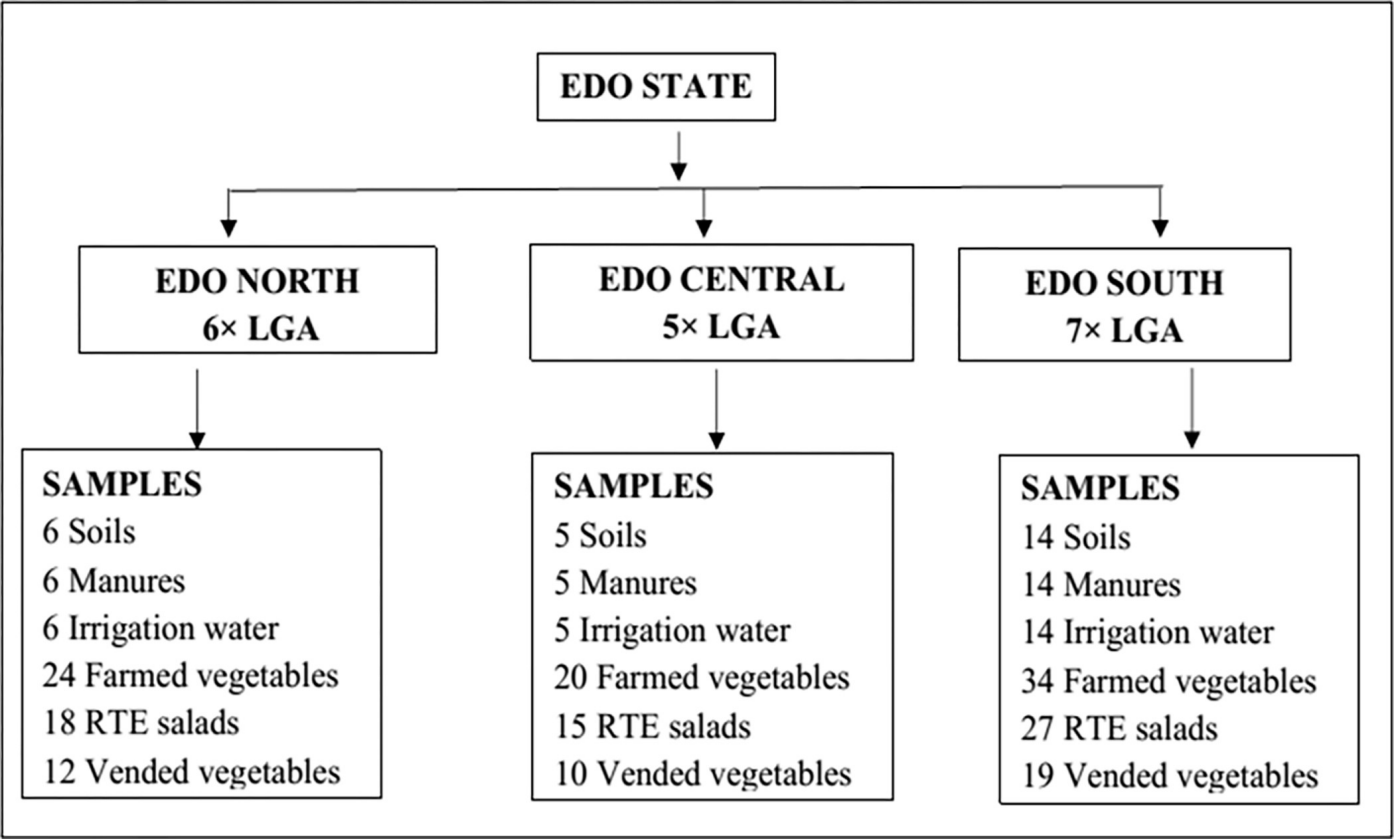
A schematic diagram of the distribution of samples from the different senatorial districts.

**Fig 2:**
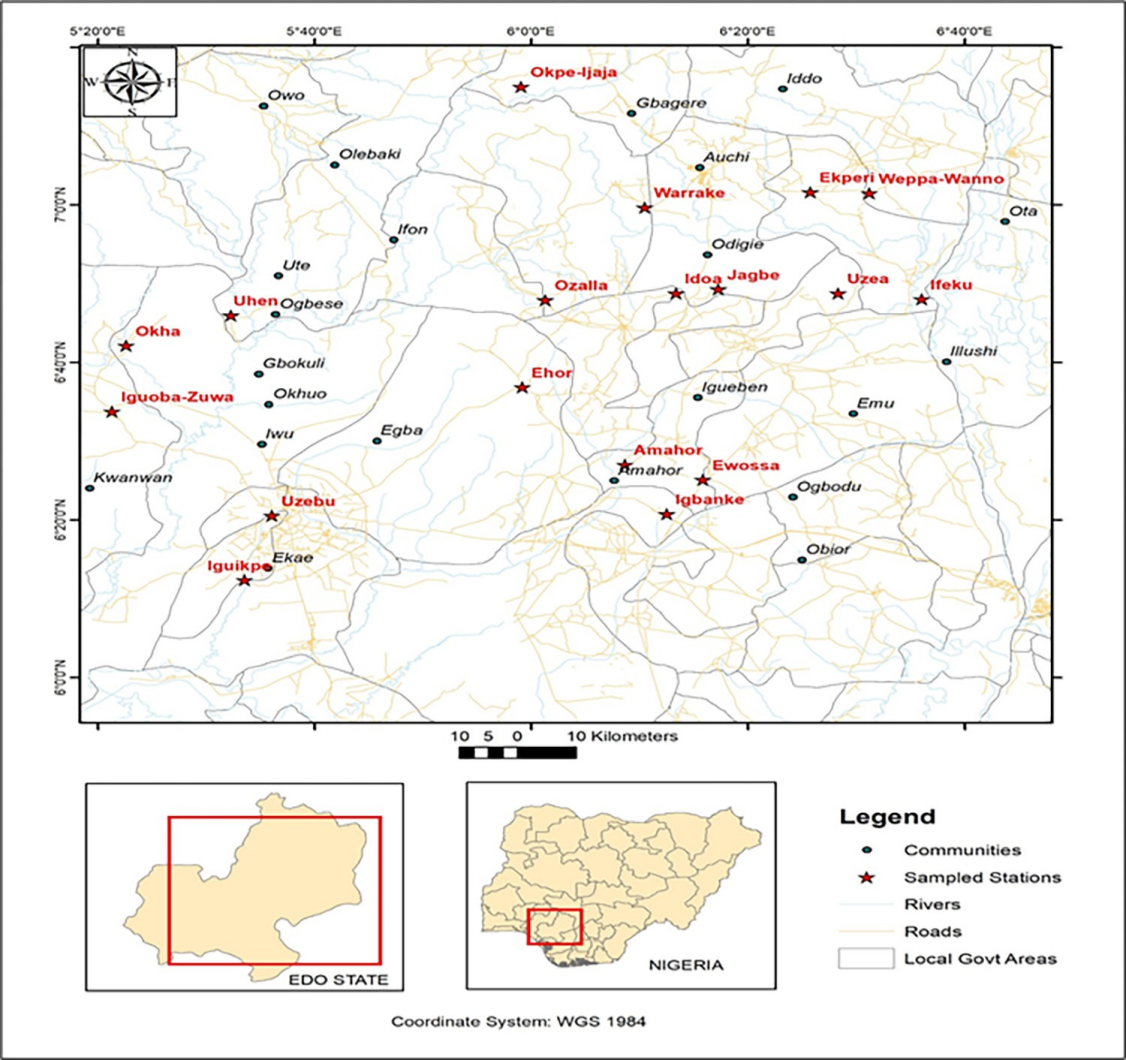
Map of the study area.

Z_1-α/2_ = Standard normal variant at 5% type I error (P < 0.05); P = Expected prevalence of 4.0–10.12% was based on previous studies [7,21,28–33]; d = Absolute error or precision (which is 5%). The expected minimum sample to be collected ranged from 59–140. Edo State was selected to pilot the study due to its strategic location in Southern Nigeria and its large-scale cultivation of vegetables due to its conducive climate and environmental conditions. There are eighteen Local Government Areas (LGA) in Edo State, and samples were collected in each LGA (Figs 1 and 2). A total of 254 samples were examined. Edo South is the most populated among the three senatorial districts and is also where the state capital (Benin City) is located (Fig 1). LGA are geographical entities within a state in Nigeria which largely depend on the land mass of the states, and Local Government Councils administer these. The senatorial districts in Nigeria consist of a combination of LGA within a state, with each state consisting of three senatorial districts. A single representative farm was surveyed from each LGA, totaling 18 farms. $Sample\;(N)=\frac{\left(Z_{1-\propto /2}{)}^{2}P(1-P\right)}{d^{2}}$ Samples from agricultural farms include soil (*n* = 25), manure (*n* = 25), irrigation water (*n* = 25), and vegetables (*n* = 78). Samples from vendors and open markets include ready-to-eat (RTE) salads (*n* = 60) and vegetables potentially consumed uncooked (*n* = 41). The fresh produce from vendors and open markets potentially consumed uncooked included: lettuce (*n* = 7), carrot (*n* = 7), tomato (*n* = 7), cabbage (*n* = 6), pepper (*n* = 7) and cucumber (*n* = 7). Ethical approval was not obtained before sampling collection since samples were not obtained directly from humans or food-producing animals. However, verbal informed consent and permission were obtained from the farm owners before sampling collection from respective farms.

### ESBL enumeration, isolation and identification

The use of the CHROMagar ESBL media for ESBL-*E*. *coli* enumeration and isolation were in line with previous studies [34,35]. Samples were processed by homogenizing 25 g of soil or vegetable samples or mixing 25 ml of irrigation water with 225 ml of buffered peptone water, diluted serially, followed by incubation for 24 h at 37°C. After that, one ml was spread plated onto CHROMagar ESBL (75006 Paris, France) media and plates were incubated for 18–24 h at 37°C. ESBL production was determined by characteristic colonial morphology and growth on culture media. Dark pink to reddish colonies on CHROMagar ESBL plate designated ESBL-producing *E*. *coli*. The cell counts of ESBL *E*. *coli* from CHROMagar ESBL were expressed in CFU/g (for vegetable, soil, and animal manure samples) or CFU/100ml (for water samples). Two to three colonies were picked per positive sample and assayed for ESBL phenotypically using the double-disk test [36]. Ceftazidime (30 μg) and ceftazidime-clavulanate (30/10 μg), as well as cefotaxime (30 μg) and cefotaxime-clavulanate (30/10 μg), were used in a standard disk diffusion procedure and incubated at 37°C for 18 h. A ≥5-mm increase in inhibition diameter for either tested antimicrobial agent in combination with clavulanate vs the zone diameter of the agent when tested alone was considered ESBL [36]. Polymerase chain reaction (PCR) was used to confirm the identity of the isolates as *E*. *coli* by targeting the *uidA* gene via specific primers [37]. A total of 64 isolates were confirmed and stored for further characterization.

### Antimicrobial susceptibility testing

The antimicrobial susceptibility of the isolates was determined using the Kirby-Bauer disk-diffusion method following recommendations outlined by the Clinical and Laboratory Standards Institute antimicrobial (CLSI) [36]. Briefly, the purified isolates were inoculated in 5.0 mL Mueller-Hinton Broth (MHB) (Lab M, Lancashire, United Kingdom) and incubated overnight. The optical density (OD) of the turbidity of the broth was adjusted to 0.5 McFarland standard, equivalent to 1.5×10^8^ cfu/mL. The McFarland turbidity standard was prepared following an established procedure [36]. Using a sterile swab stick, respective broth cultures were aseptically swabbed on Mueller Hinton Agar (Lab M, Lancashire, United Kingdom). The antimicrobial disks used (Mast Diagnostics, UK) included: Cephems (cefpodoxime 10 μg, cefotaxime 30 μg, ceftazidime 30 μg, ceftriaxone 30 μg); monobactams (aztreonam 30 μg), penicillins (ampicillin 10 μg, piperacillin 100 μg); β-lactam combination agents (ampicillin-sulbactam 10/10 μg, amoxicillin-clavulanate 20/10 μg, piperacillin-tazobactam 100/10 μg, meropenem-vaborbactam 20/10 μg, ceftazidime-avibactam 30/20 μg); carbapenems (meropenem 10 μg, ertapenem 10 μg, imipenem 10 μg); aminoglycosides (gentamicin 10 μg, kanamycin 30 μg, streptomycin 10 μg), tetracyclines (tetracycline 30 μg, doxycycline 30 μg); quinolones and fluoroquinolones (levofloxacin 5 μg, ofloxacin 5 μg, ciprofloxacin 5 μg); folate pathway antagonists (trimethoprim-sulfamethoxazole 1.25/23.75 μg), nitrofurans (nitrofurantoin 300 μg) and phenicols (chloramphenicol 30 μg) [36]. Isolates were classified as showing a resistant, intermediate or susceptible phenotype according to CLSI standards [36]. The multiple antibiotic resistance index (MARI) was determined to be the total number of antimicrobials the organism is resistant to divided by the total number of antimicrobials the organism was subjected to [38].

### Characterization of ESBL *E*. *coli* isolates regarding ESBL determinants and other antibiotic resistance genes

All CHROMagar positive, ESBL-producing presumptive *E*. *coli* isolates that were confirmed by PCR as *E*. *coli*, were screened for genetic element determinants of ESBL resistance, i.e., *bla*_CTX−M−15,_ *bla*_TEM_, *bla*_SHV_ and all CTX-M-groups (*bla*_CTX−M−1−group_, *bla*_CTX−M−2−group_, *bla*_CTX−M−9−group_, *bla*_CTX−M−8−group,_ *bla*_CTX−M−14−group_), as well as the *bla*_OXA−1−group_. The *bla*_OXA−47_, *bla*_NDM−1_ (carbapenem-resistance genes), and *bla*_CMY−2_ genes (AmpC lactamase) were PCR amplified using conditions as described previously [39]. The presence of resistance determinants for tetracycline [*tet*A, *tet*B, *tet*M], sulfonamides [*sul*1, *sul*2 and*sul*3], aminoglycosides [*ant(4´)-Ia*, *aacC*(3)-1], quinolones [*qnr*A, *qnr*B, *qnr*C and *qnr*S] and chloramphenicol [*cat*1, *cat*2, *cat*3] resistance were also determined [40]. As reported before, intI2 and intI1 genes (encoding class 2 and class 1 integrases, respectively) were also investigated [40]. Single colonies of individual isolates were aerobically cultivated in Tryptone Soy Broth (TSB) (Lab M, Lancashire UK) in a shaker incubator at 200 rpm at 30°C for 24 h. The cells were pelleted via centrifugation (12,500 ×g for 10 min). Deoxyribonucleic acid (DNA) was extracted using the peqGOLD Bacterial DNA Kit (Peqlab Biotechnologie GmbH, Germany), following the manufacturer’s protocols. PCR was performed using a Peltier-based thermal cycler (MG963/Y, Hangzhou, Zhejiang, China) with thermocycling conditions and specific primers for each of the determinants as described in S1 Table.

### Data analysis

The data from this study were analyzed using Microsoft Excel 2013 and SPSS 21.0. The prevalence and occurrence from this study were expressed in percentage (%) while the population density from the samples was expressed as mean ± standard deviation of the mean.

## Results

### Prevalence of ESBL *E*. *coli* from agricultural farms and open markets

A total of 64 *E*. *coli* isolates were identified using PCR and further characterized (Table 1). In total, 68% (17/25) of ESBL *E*. *coli* were isolated from agricultural farm soil, 84% (21/25) from manure, 28%(7/25) from irrigation water and 24.4% (19/78) from vegetables, while 20% (12/60) were obtained from RTE salads from vendors and open markets and 36.6% (15/41) from vegetables that can be consumed uncooked which were also obtained from vendors and open markets. Amongst the vegetables from vendors which can be consumed uncooked, the tomato had a higher prevalence of 100% (7/7) than cabbage 66.7% (4/6), lettuce 42.9% (3/7) and pepper 14.3% (1/7). Amongst the vegetables from farms, the fluted pumpkin had the highest prevalence at 26.3% (5/19), followed by Lagos spinach at 21.1% (4/19), African spinach at 21.1% (4/19), waterleaf at 15.8% (3/19), jute leaves 10.5% (2/19) and curry leaf 5.3% (1/19). The mean counts of ESBL E. coli based on the occurrence of reddish to dark pink colonies on the CHROMagar ESBL plate were 2.72 × 10^3^ ± 0.76 CFU/g for soil, 7.68 × 10^3^ ± 1.49 CFU/g for manure, 1.13 × 10^2^ ± 0.35 CFU/mL for irrigation water, 1.25 × 10^2^ ± 0.26 CFU/g for vegetables, 2.51 × 10^1^ ± 0.31 CFU/g for RTE salads and 9.4 × 10^1^ ± 0.22 CFU/g for vegetables potentially consumed uncooked.

**Table 1:** Prevalence of ESBL *E*. *coli* from agricultural farms and open markets.

Sampling locations	Samples	Mean population cell density	Prevalence n(%)	ESBL *E*. *coli* recovered (*n* = 64)
Samples from agricultural farms	Soil (*n* = 25)	2.72 × 10^3^ ±0.76 CFU/g	17 (68%)	ESBL *E*. *coli* (*n* = 11)
	Manure (*n* = 25)	7.68 × 10^3^ ±1.49 CFU/g	21 (84%)	ESBL *E*. *coli* (*n* = 13)
	Irrigation water (*n* = 25)	1.13 × 10^2^ ±0.35 CFU/mL	7 (28%)	ESBL *E*. *coli* (*n* = 5)
	Vegetables (*n* = 78)	1.25 × 10^2^ ±0.26 CFU/g	19 (24.4%) Fluted Pumpkin 5(26.3%) Jute leaves 2(10.5%) Curry leaf 1 (5.3%) Waterleaf 3(15.8%) Lagos spinach 4(21.1%) African spinach 4(21.1%)	ESBL *E*. *coli* (*n* = 15) Fluted pumpkin (*n* = 4) Jute leaves (*n* = 1) Curry leaf (*n* = 1) Waterleaf (*n* = 2) Lagos spinach (*n* = 4) African spinach (*n* = 3)
Samples from vendors and open markets	Ready-to-eat (RTE) salads (*n* = 60)	2.51 × 10^1^ ± 0.31 CFU/g	12 (20%)	ESBL *E*. *coli* (*n* = 7)
	Vegetables potentially consumed uncooked (*n* = 41) [lettuce (*n* = 7), carrot (*n* = 7), tomato (*n* = 7), cabbage (*n* = 6), pepper (*n* = 7) and cucumber (*n* = 7)]	9.4 × 10^1^ ± 0.22 CFU/g	15 (36.6%) Lettuce 3 (42.9%) Carrot 0 Tomato 7 (100%) Cabbage 4 (66.7%) Pepper 1 (14.3%) Cucumber 0	ESBL *E*. *coli* (*n* = 13) Lettuce (*n* = 3) Tomato (*n* = 6) Cabbage (*n* = 3) Pepper (*n* = 1)

**Key:** ESBL: Extended spectrum β-lactamase.

### Antimicrobial susceptibility profile of the ESBL *E*. *coli* isolates

ESBL *E*. *coli* isolates showed the following incidences of resistance towards 26 antibiotics (Table 2): ceftazidime and cefotaxime 100% (64/64); cefpodoxime 96.9% (62/64); ceftriaxone 98.4% (63/64); amoxicillin-clavulanate 50% (32/64); ciprofloxacin and ampicillin 48.4% (31/64); doxycycline 45.3% (29/64); piperacillin 43.8% (28/64); kanamycin 42.2% (27/64); ofloxacin and gentamicin 37.5% (24/64); levofloxacin and trimethoprim-sulfamethoxazole 35.9% (23/64); chloramphenicol 26.6% (17/64); ampicillin-sulbactam 14.1% (9/64); ceftazidime-avibactam 6.3% (4/64). All the isolates were sensitive to meropenem-vaborbactam, nitrofurantoin, ertapenem, imipenem and meropenem.

**Table 2:** Prevalence of antibiotic resistance profile of the isolates.

Antimicrobial class	Antibiotics	ESBL *E*. *coli* (*n* = 64)		
		Resistance n(%)	Intermediate n(%)	Sensitive n(%)
Cephems	CEP	62 (96.9)	2 (3.1)	0
	CAZ	64 (100)	0	0
	CTX	64 (100)	0	0
	CEF	63 (98.4)	1 (1.6)	0
Monobactams	AZM	25 (39.1)	11 (17.2)	28 (43.8)
Penicillins	AMP	31 (48.4)	17 (26.6)	16 (25)
	PIP	28 (43.8)	11 (17.2)	25 (39.1)
β-lactam combination agents	AMC	32 (50)	9 (14.1)	23 (35.9)
	AMS	9 (14.1)	11 (17.2)	44 (68.8)
	CEA	4 (6.3)	7 (10.9)	53 (82.8)
	MEV	0	0	64 (100)
	PIT	9 (14.1)	12 (18.8)	43 (67.2)
Carbapenems	ETP	0	0	64 (100)
	IMI	0	0	64 (100)
	MEM	0	0	64 (100)
Aminoglycosides	GEN	24 (37.5)	17 (26.6)	23 (35.9)
	KAN	27 (42.2)	14 (21.9)	23 (35.9)
	STR	32 (50)	12 (18.8)	20 (31.3)
Tetracyclines	TET	33 (51.6)	6 (9.4)	25 (39.1)
	DOX	29 (45.3)	8 (12.5)	27 (42.2)
Quinolones and fluoroquinolones	CIP	31 (48.4)	17 (26.6)	16 (25)
	LEV	23 (35.9)	19 (29.7)	22 (34.4)
	OFL	24 (37.5)	22 (34.4)	18 (28.1)
Folate pathway antagonists	STX	23 (35.9)	14 (21.9)	27 (42.2)
Phenicols	CHL	17 (26.6)	12 (18.8)	35 (54.7)
Nitrofurans	NIT	0	0	64 (100)

CEP: Cefpodoxime (10 μg), CAZ: Ceftazidime (30 μg), CTX: Cefotaxime (30 μg), CEF: Ceftriaxone (30 μg), AZM: Aztreonam (30 μg), AMP: Ampicillin (10 μg), PIP: Piperacillin (100 μg), AMC: Amoxicillin-clavulanate (20/10 μg), AMS: Ampicillin-sulbactam (10/10 μg), CEA: Ceftazidime-avibactam (30/20 μg), MEV: Meropenem-vaborbactam (20/10 μg), PIT: Piperacillin-tazobactam (100/10 μg), ETP: Ertapenem (10 μg), IMI: Imipenem (10 μg), MEM: Meropenem (10 μg), GEN: Gentamicin (10 μg), KAN: Kanamycin (30 μg), STR: Streptomycin (10 μg), DOX: Doxycycline (30 μg), TET: Tetracycline (30 μg), CIP: Ciprofloxacin (5 μg), LEV: Levofloxacin (5 μg), OFL: Ofloxacin (5 μg), CHL: Chloramphenicol (30 μg), STX: Trimethoprim-sulfamethoxazole (1.25/23.75 μg), NIT: Nitrofurantoin (300 μg), value in parathesis represent percentage (%).

A total of 85.9% (55/64) isolates were resistant to ≥3 and ≤7 antimicrobial classes, which categorizes these as being multidrug-resistant (MDR). The multiple antibiotic resistance (MAR) index of the isolates ranged from 0.27 –to 0.62 for these 55 MDR isolates. However, all the isolates were multi-resistant (resistant to a minimum of 4 antibiotics) and displayed 52 resistance phenotypes (Table 3). A MAR index >0.5 was observed to be associated with isolates recovered from the agricultural environments. In addition, isolates from agricultural environments seemingly showed a higher resistance phenotype than isolates from vendors and open markets. The phenotypic resistance phenotype of the isolates was independent of the beta-lactamase determinants of the isolates (Table 3).

**Table 3:** Multiple antibiotic resistance profiles of the ESBL *E*. *coli* isolates.

Isolate code	Isolate source	Antimicrobial resistance phenotype	MARI	N° of AMR Class	Beta- lactamase genes	Other resistance Determinants
S02	Soil	CEP, CAZ, CTX, CEF, AZM, AMP, PIP, STR, TET, STX	0.38	6	*bla*_CTX−M−1,_ *bla*_TEM_	*tet*M, *sul*2
S03	Soil	CEP, CAZ, CTX, CEF, AZM, AMC, GEN, KAN, STR, TET, DOX, STX	0.46	6	*bla*_CTX−M−1,_ *bla*_TEM_	*tet*A, *tet*M, *ant(4´)-Ia*, *int*I1
S07	Soil	CEP, CAZ, CTX, CEF, AZM, AMP, PIP, TET, DOX, STX	0.38	5	*bla*_CTX−M−1,_ *bla*_TEM_	*tet*A, *tet*M
S08	Soil	CEP, CAZ, CTX, CEF, AMP, PIP, AMC, STR, TET, DOX, STX	0.42	6	*bla*_TEM_	*tet*M, *sul*2
S11	Soil	CEP, CAZ, CTX, CEF, AZM, AMP, AMC, TET, STX, CHL	0.38	7	*bla*_CTX−M−1,_ *bla*_TEM_	*tet*M, *cat*::*p*C194
S12	Soil	CEP, CAZ, CTX, CEF, AMC, CEA, GEN, KAN, STR, OFL, CHL	0.42	5	*bla*_CTX−M−1,_ *bla*_TEM_	*ant(4´)-Ia*, *qnr*B, *cat*::*p*C194, *int*I1
S13	Soil	CEP, CAZ, CTX, CEF, AZM, AMP, PIP, TET, DOX, STX	0.38	5	*bla*_CTX−M−1_	*tet*A, *tet*M, *sul*2
S17	Soil	CEP, CAZ, CTX, CEF, AMP, PIP, AMC, TET, DOX, CIP, OFL, STX	0.46	6	*bla*_CTX−M−15,_ *bla*_CTX−M−1,_ *bla*_TEM_	*sul*2, *qnr*A, *int*I1
S19	Soil	CEP, CAZ, CTX, CEF, AMP, PIP, AMC, GEN, KAN, STR, TET, DOX, STX	0.5	6	*bla*_CTX−M−1,_ *bla*_TEM_	*sul*3, *ant(4´)-Ia*, *int*I1
S23	Soil	CEP, CAZ, CTX, CEF, AZM, AMC, GEN, KAN, STR, TET, DOX, STX	0.46	6	*bla*_TEM_	*sul*2, *int*I1
S24	Soil	CEP, CAZ, CTX, CEF, AZM, AMP, PIP, AMS, TET, DOX, CIP, OFL, STX	0.5	7	*bla*_CTX−M−1_	*sul*2, *qnr*A, *int*I2
M01	Manure	CEP, CAZ, CTX, CEF, AMP, PIP, AMC, STX, CHL	0.35	5	*bla*_CTX−M−1_	*sul*3, *cat*::*p*C194
M04	Manure	CEP, CAZ, CTX, CEF, AZM, AMC, CEA, GEN, KAN, STR, TET, DOX, STX	0.5	6	*bla*_CTX−M−1,_ *bla*_TEM_	*sul*3, *ant(4´)-Ia*, *int*I1
M05	Manure	CEP, CAZ, CTX, CEF, AZM, AMP, AMC, GEN, KAN, STR, TET, DOX	0.46	6	*bla*_CTX−M−1_	*tet*A, *tet*M
M07	Manure	CEP, CAZ, CTX, CEF, AZM, AMP, PIP, AMS, PIT, TET, DOX, STX	0.46	6	*bla*_CTX−M−1,_ *bla*_TEM_	*sul*2
M09	Manure	CEP, CAZ, CTX, CEF, AMP, PIP, AMC, GEN, KAN, STR, CIP, LEV, OFL, STX, CHL	0.58	7	*bla*_TEM_	*sul*1, *qnr*A, *cat*::*p*C194, *int*I2
M10	Manure	CEP, CAZ, CTX, CEF, AZM, AMC, GEN, KAN, STR, TET, DOX, CIP, OFL, LEV	0.54	6	*bla*_CTX−M−1,_ *bla*_TEM_	*tet*B, *tet*M, *qnr*A, *int*I1
M12	Manure	CEP, CAZ, CTX, CEF, AZM, AMP, AMS, PIT, TET, DOX	0.38	5	*bla*_CTX−M−1_	*sul*2
M15	Manure	CEP, CAZ, CTX, CEF, AMP, PIP, AMC, CEA, STR, TET, OFL	0.42	6	*bla*_CTX−M−15,_ *bla*_CTX−M−1_	*qnrC*
M16	Manure	CEP, CAZ, CTX, CEF, AMC, GEN, KAN, STR, TET, DOX, CIP, OFL, LEV	0.5	5	*bla*_TEM_	*qnr*A
M20	Manure	CEP, CAZ, CTX, CEF, AZM, AMP, PIP, AMC, PIT, GEN, KAN, STR, TET, DOX, STX, CHL	0.62	7	*bla*_CTX−M−1_	*tet*A, *tet*M, *sul*2, *sul*3, *ant(4´)-Ia*
M23	Manure	CEP, CAZ, CTX, CEF, AZM, AMP, PIP, AMS, GEN, KAN, STR, CIP, OFL, CHL	0.54	7	*bla*_CTX−M−1,_ *bla*_TEM_	*qnr*A, *cat*::*p*C194
M24	Manure	CEP, CAZ, CTX, CEF, AZM, AMC, TET, DOX, CIP, OFL, CHL	0.42	6	*bla*_CTX−M−1_	*tet*M, *qnr*B, *cat*::*p*C223
M25	Manure	CEP, CAZ, CTX, CEF, AMP, PIP, AMC, CEA, TET, DOX, CIP, OFL, LEV	0.5	5	*bla*_CTX−M−1_	*tet*M, *qnrC*, *int*I1
IW04	Irrigation water	CEP, CAZ, CTX, CEF, AZM, AMP, PIP, AMC, PIT, GEN, KAN, STR, CIP, LEV, OFL	0.58	6	*bla*_CTX−M−1,_ *bla*_TEM_	*ant(4´)-Ia*, *qnr*A
IW07	Irrigation water	CEP, CAZ, CTX, CEF, AZM, AMP, PIP, AMS, GEN, KAN, STR, TET, DOX, STX	0.54	7	*bla*_CTX−M−1_	*tet*M, *aacC*(3)-1
IW11	Irrigation water	CEP, CAZ, CTX, CEF, AZM, AMP, PIP, AMC, TET, DOX, CIP, LEV, OFL, CHL	0.54	7	*bla*_CTX−M−15,_ *bla*_CTX−M−1_	*tet*B, *tet*M, *qnrC*, *cat*::*p*C194, *int*I1
IW16	Irrigation water	CEP, CAZ, CTX, CEF, AZM, PIT, GEN, KAN, STR, CIP, LEV, OFL, STX	0.5	6	*bla*_CTX−M−1,_ *bla*_TEM_	*sul*2, *ant(4´)-Ia*, *qnr*A
IW22	Irrigation water	CEP, CAZ, CTX, CEF, AZM, PIT, GEN, KAN, STR, TET, DOX, CIP, OFL, LEV, CHL	0.58	7	*bla*_CTX−M−1,_ *bla*_TEM_	*tet*A, *qnrC*, *cat*::*p*C221, *int*I1
FP02	Fluted pumpkin	CEP, CAZ, CTX, CEF, AMP, PIP, GEN, KAN, STR, CIP, OFL	0.42	4	*bla*_TEM_	*sul*1, *aacC*(3)-1
FP65	Fluted pumpkin	CEP, CAZ, CTX, CEF, AMC, KAN, STR, TET, DOX, CIP, LEV, OFL	0.46	5	*bla*_CTX−M−1,_ *bla*_TEM_	*tet*M, *qnrC*
FP72	Fluted pumpkin	CEP, CAZ, CTX, CEF, AMC, GEN, KAN, STR, TET, DOX, STX	0.42	5	*bla*_CTX−M−1_	*tet*A, *tet*M, *sul*2, *aacC*(3)-1
FP76	Fluted pumpkin	CEP, CAZ, CTX, CEF, AZM, AMC, TET, DOX, CIP, LEV, OFL, CHL	0.46	6	*bla*_TEM_	*tet*M, *cat*::*p*C223, *int*I1
JL23	Jute leaves	CEP, CAZ, CTX, CEF, AMS, PIT, TET, DOX, CIP, LEV	0.38	4	*bla*_CTX−M−1,_ *bla*_TEM_	*tet*M, *qnrC*
CL15	Curry leaf	CEP, CAZ, CTX, CEF, AMC, STR, TET, DOX, CHL	0.35	5	*bla*_CTX−M−1,_ *bla*_TEM_	*tet*A, *tet*M, *cat*::*p*C221
WL28	Waterleaf	CEP, CAZ, CTX, CEF, AMP, PIP, GEN, KAN, STR, CIP, LEV, OFL	0.46	4	*bla*_TEM_	*qnr*S, *int*I1
WL07	Waterleaf	CEP, CAZ, CTX, CEF, AMC, PIT, GEN, KAN, STR, CHL	0.38	4	*bla*_CTX−M−1_	*aacC*(3)-1, *cat*::*p*C221
LS11	Lagos spinach	CEP, CAZ, CTX, CEF, AZM, CIP, LEV, STX	0.31	4	*bla*_CTX−M−1_	*sul*1, *qnr*S
LS17	Lagos spinach	CEP, CAZ, CTX, CEF	0.15	1	*bla*_CTX−M−1_	-
LS25	Lagos spinach	CEP, CAZ, CTX, CEF	0.15	1	*bla*_CTX−M−1_	-
LS61	Lagos spinach	CEP, CAZ, CTX, CEF, AMP, PIP, TET, DOX, CHL	0.35	4	*bla*_TEM_	*tet*A, *cat*::*p*C221
AS54	African spinach	CEP, CAZ, CTX, CEF, AMC, PIT, GEN, KAN, STR, TET, CIP, OFL, CHL	0.5	6	*bla*_CTX−M−1_	*tet*M, *aacC*(3)-1
AS43	African spinach	CEP, CAZ, CTX, CEF	0.15	1	*bla*_CTX−M−1_	-
AS39	African spinach	CEP, CAZ, CTX, CEF, AMC, KAN, STR, TET, DOX, CIP, LEV, OFL	0.46	5	*bla*_CTX−M−1_	*tet*B
RAS13	RTE salads	CEP, CAZ, CTX, CEF, AMS, CIP, LEV, STX	0.31	4	*bla*_CTX−M−1_	*sul*1
RAS19	RTE salads	CAZ, CTX, CEF, AZM, AMP, PIP, CHL	0.27	4	*bla*_CTX−M−15,_ *bla*_CTX−M−1_	*cat*::*p*C221
RAS31	RTE salads	CEP, CAZ, CTX, CEF, AMC, CIP, LEV	0.27	3	*bla*_CTX−M−1_	*qnr*S
RAS42	RTE salads	CEP, CAZ, CTX, CEF, AZM, AMP, PIP, TET, DOX	0.35	4	*bla*_CTX−M−1,_ *bla*_TEM_	*tet*M
RAS43	RTE salads	CEP, CAZ, CTX, CEF, AMP, PIP, CIP, LEV, OFL, STX	0.38	4	*bla*_CTX−M−1_	*sul*1
RAS49	RTE salads	CEP, CAZ, CTX, CEF, AMC, TET, DOX, CIP, OFL	0.35	4	*bla*_CTX−M−15,_ *bla*_TEM_	*tet*M, *qnr*S
RAS53	RTE salads	CAZ, CTX, AZM, AMP, PIP, GEN, KAN, STR, CIP, LEV	0.38	5	*bla*_CTX−M−1_	*aacC*(3)-1
LE07	Lettuce	CEP, CAZ, CTX, CEF, AMC, CIP, LEV, CHL	0.31	4	*bla*_TEM_	*qnr*S, *cat*::*p*C221
LE08	Lettuce	CEP, CAZ, CTX, CEF	0.15	1	-	-
LE11	Lettuce	CEP, CAZ, CTX, CEF, AMP, PIP, AMS, CIP, LEV, STX	0.38	5	*bla*_CTX−M−1_	*sul*1
TO16	Tomato	CEP, CAZ, CTX, CEF, AMC, GEN, KAN, STR, CIP, OFL	0.38	4	*bla*_CTX−M−15,_ *bla*_TEM_	*aacC*(3)-1
TO17	Tomato	CEP, CAZ, CTX, CEF	0.15	1	*bla*_CTX−M−1_	-
TO19	Tomato	CEP, CAZ, CTX, CEF	0.15	1	-	-
TO20	Tomato	CEP, CAZ, CTX, CEF	0.15	1	*bla*_CTX−M−1_	-
TO21	Tomato	CEP, CAZ, CTX, CEF, AMP, PIP, AMS, STR, CIP, LEV	0.38	5	*bla*_CTX−M−15,_ *bla*_TEM_	*qnr*S
TO29	Tomato	CEP, CAZ, CTX, CEF, AMP, PIP, GEN, KAN, STR	0.35	3	*bla*_TEM,_ *bla*_TEM_	*aacC*(3)-1
CA37	Cabbage	CEP, CAZ, CTX, CEF, AMC, KAN, STR, CIP, LEV, STX, CHL	0.42	6	*bla*_CTX−M−1_	*sul*1, *cat*::*p*C221, *int*I1
CA39	Cabbage	CEP, CAZ, CTX, CEF	0.15	1	*bla*_CTX−M−1_	-
CA40	Cabbage	CEP, CAZ, CTX, CEF, AMP, PIP, AMC, GEN, KAN, STR, CIP, LEV, OFL	0.5	5	*bla*_CTX−M−1_	*aacC*(3)-1, *qnr*S
PE03	Pepper	CEP, CAZ, CTX, CEF	0.15	1	*bla*_CTX−M−1_	-

CEP: Cefpodoxime (10 μg), CAZ: Ceftazidime (30 μg), CTX: Cefotaxime (30 μg), CEF: Ceftriaxone (30 μg), AZM: Aztreonam (30 μg), AMP: Ampicillin (10 μg), PIP: Piperacillin (100 μg), AMC: Amoxicillin-clavulanate (20/10 μg), AMS: Ampicillin-sulbactam (10/10 μg), CEA: Ceftazidime-avibactam (30/20 μg), MEV: Meropenem-vaborbactam (20/10 μg), PIT: Piperacillin-tazobactam (100/10 μg), ETP: Ertapenem (10 μg), IMI: Imipenem (10 μg), MEM: Meropenem (10 μg), GEN: Gentamicin (10 μg), KAN: Kanamycin (30 μg), STR: Streptomycin (10 μg), DOX: Doxycycline (30 μg), TET: Tetracycline (30 μg), CIP: Ciprofloxacin (5 μg), LEV: Levofloxacin (5 μg), OFL: Ofloxacin (5 μg), CHL: Chloramphenicol (30 μg), STX: Trimethoprim-sulfamethoxazole (1.25/23.75 μg), NIT: Nitrofurantoin (300 μg).

### Presence of resistance determinants in ESBL *E*. *coli* isolates

**Fig 3:**
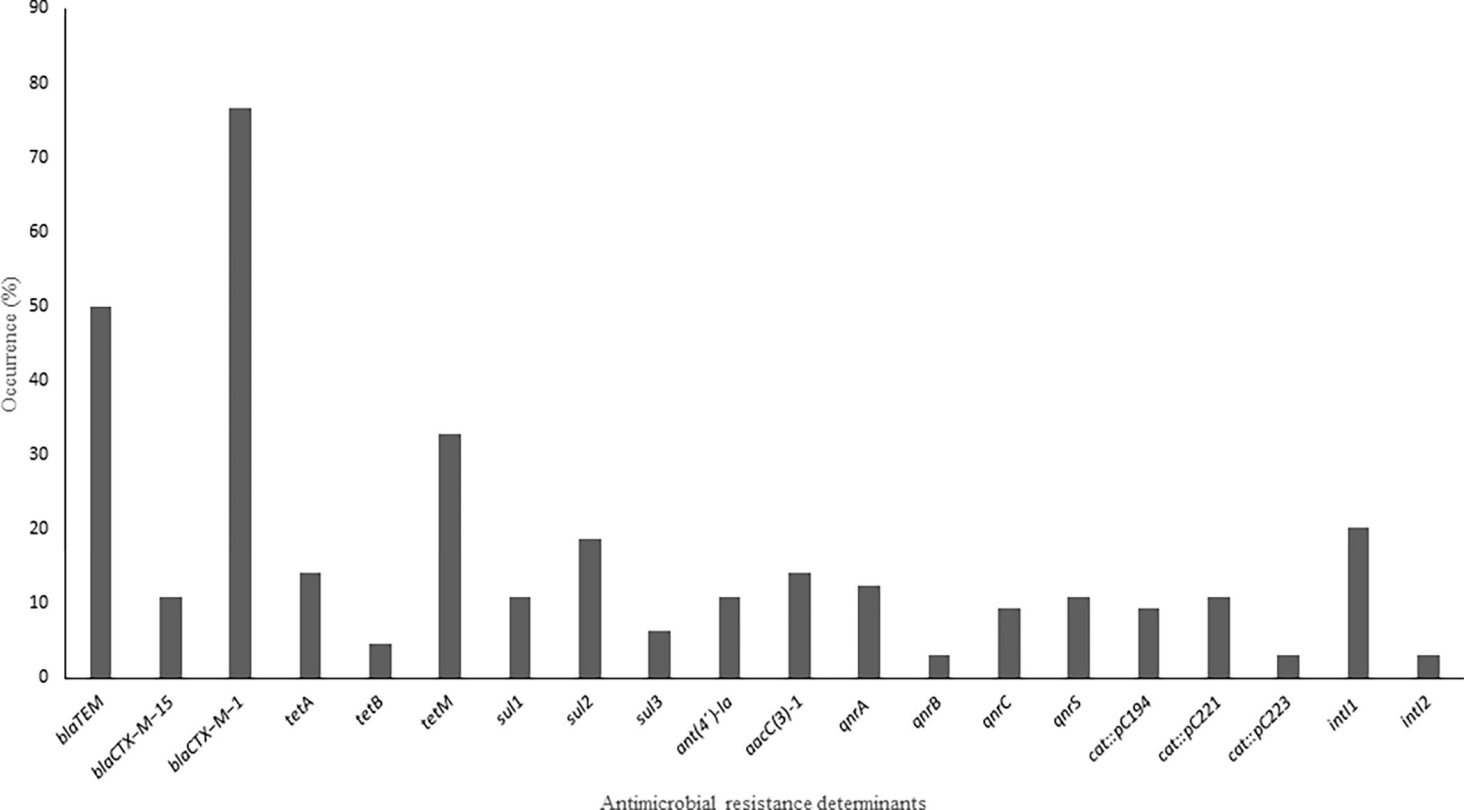
Occurrence of antimicrobial resistance determinants from the ESBL *E*. *coli* isolates.

The proportions of ESBL *E*. *coli* isolates that harboured beta-lactamase genes in Fig 3 include *bla*_TEM_ 50% (32/64), *bla*_CTX−M−15_ 10.9% (7/64) and *bla*_CTX−M−1_ 76.6% (49/64). The proportions of ESBL *E*. *coli* isolates harbouring other resistance determinants include *tet*A 14.1% (9/64), *tet*B 4.7% (3/64), *tet*M 32.8% (21/64), *sul*1 10.9% (7/64), *sul*2 18.8% (11/64), *sul*3 6.3% (4/64), *ant(4´)-Ia* 10.9% (7/64), *aacC*(3)-1 14.1% (9/64), *qnr*A 12.5% (8/64), *qnr*B 3.1% (2/64), *qnrC* 9.4% (6/64), *qnr*S 10.9% (7/64), *cat*::*p*C194 9.4% (6/64), *cat*::*p*C221 10.9% (7/64), *cat*::*p*C223 3.1% (2/64), *int*I1 20.3% (13/64), *int*I2 3.1% (2/64). The 55 MDR isolates harboured a minimum of 1 and a maximum of 5 AMR determinants. The 55 MDR isolates also harboured a minimum of 1 and a maximum of 3 beta-lactamase determinants. The detection of the beta-lactamase determinants of the isolates was independent of the detection of other antibiotic resistance genes. All isolates harbouring respective antibiotic resistance genes showed phenotypic resistance to ≥1 antibiotic from the antimicrobial class (Table 3).

## Discussion

Vegetables are highly prone to contamination by microorganisms through contact with soil, water and handling during harvest or after-harvest processing. They may therefore harbour a diverse range of bacterial pathotypes, including human and plant pathogens. Quarcoo et al. [21] reported that counts expressed in CFU/g ranged from 186 to 3000, with the highest counts found in lettuce, with all lettuce samples positive for *E*. *coli*. Though the population density from Quarcoo et al. [21] study is similar to ours, the occurrence rate is higher than ours. This could be attributed to the selective characteristics of the media used in our research. Reported microbial load range of <3 to >1100 MPN/g from Vegetables in Malaysia [41] and 2.31 ±0.86 log CFU/g to 5.50 ±0.7 log CFU/g in Spain [31] were also similar to those of our study. The total counts of ESBL-*E*. *coli* from the farm produce and environments were determined to evaluate their microbiological quality and safety. The potential sources of ESBL-*E*. *coli* contamination could result from faecal contamination, poor water quality, poor distribution and sanitary conditions, and poor handling practices. Lower ESBL-*E*. *coli* prevalence from farmed vegetables compared to those of our study have been reported from Finland, Spain, Germany, Southern Thailand, and the United States [31,42–46]. A higher prevalence of 62.11% (59/95) has been reported in Malaysia [41]. Zurfluh et al. [47] documented that 25.7% of retail store samples determined were positive for ESBL isolates, which was more comparable to the results of this study. Soré et al. [48] reported that 42.47% of salad samples were positive for ESBL-producing isolates. On the other hand, that prevalence was higher than the prevalence recovered from our study, and the differences may be explained by different soils, manure and agricultural practices of the other regions/countries. Variations in the prevalence of ESBL-producing *E*. *coli* isolates depend on the sample size and types of vegetables tested. ESBL strains have been occasionally found in human infections and foods [8,49,50]. In a recent population-based modelling study, 18.9% of human ESBL-*E*. *coli* carriage was attributed to a food source [51], highlighting the importance of AMR surveillance and hygiene measures for food products.

In this study, high heterogeneity in the presence of ESBL *E*. *coli* was observed both at the farm level and from open markets. Njage and Buys [52] documented an increased incidence of ESBL-producing *E*. *coli* in irrigation water (73%-64%) samples and lettuce at harvest (90%) in South Africa. A higher prevalence was recounted from the irrigation water in this study. In the Netherlands, irrigation water (100%) and harvested lettuce (14.7%) were also found to harbour beta-lactamase-producing *Enterobacteriaceae* [7]. The incidence in irrigation water was far higher than observed in this study. Using a discrete-time model by Costa et al. [53], it was reported that ESBL-*E*. *coli* prevalence reached an equilibrium prevalence of 0.65% in the open community, where the colonization of the open community could primarily be attributed to the open community itself (62%), followed by vegetable consumption (29.5%), and contact with farmers (8.5%). A clear link between irrigation water and leafy green vegetable *E*. *coli* isolates has been established [54]. As low-income countries have higher AMR abundance in their wastewater [20,22,55,56], the risk of ESBL dissemination is greater. Using contaminated irrigation water may directly link to AMR contamination, mainly if the water is applied to edible parts of fruits and vegetables [57]. The types of nutrients that are frequently present on the leaf surface of leafy green vegetables can be categorized into two: inorganic (ions) and organic molecules (organic acids and carbohydrates) [58]. Carbohydrates (dominant phyllosphere sugars are glucose, fructose and sucrose) are of particular interest due to their capacity to readily support the growth of enteric bacteria such as *E*. *coli*. Different factors could again account for these differences, but using untreated wastewater, seasons and manure application as fertilizers might be significant factors [58]. Some authors have previously documented the identification of ESBL *E*. *coli* and the possible transmission of antibiotic-resistant bacteria via treated wastewater for irrigation [58,59], while others could not find a clear connection [60].

Concerns about the quality and safety of vegetable salads and fresh produce have increased, despite their essential nutritional benefits to humans [6–8,14,19]. Outbreaks associated with fresh produce portray a dearth of information crucial in understanding the ecology of ESBL *E*. *coli* outside animal and human-animal hosts [61]. The microbial incidences reported for fresh produce could be attributed to contaminated cultivated soil, poor storage methods and poor agricultural practices by handlers. The increased microbial occurrence on leafy greens may be linked to the structure of their surface, with many folds and furrows and a corresponding high surface area, which may lend itself to colonization. Fresh produce contamination by pathogenic bacteria is currently emerging as a significant food safety concern, and the occurrence of MDR bacteria can signify an additional hazard. Prevalence of MDR (96.1%) and resistance to aminoglycosides (46.7–66.7) in ESBL-producing *Enterobacteriaceae* have been reported previously [47,62], which is similar to the findings from this study. Richter et al. [63] reported that 98% of their isolates showed an MDR phenotype, close to the 100% of our research. Vegetables are often eaten without heating processes, leading to a greater risk of acquiring resistant bacteria [6,14,19]. Microbial food quality control maintains a critical factor in continually controlling foodborne illnesses (one of the primary causes of mortality and morbidity) coupled with a cost-intensive loss for food companies.

Contamination by ESBL *E*. *coli* of farm environments and vegetable samples from our study hypothetically could primarily be from animal sources. Since the manure used in farm environments is predominantly from animal sources. These animals are usually treated with antibiotics for therapeutic and prophylactic purposes [64,65]. These antibiotic residues could be excreted by the animal in an unmetabolized or poorly metabolized form, resulting in the development and transmission of AMR [66]. But also, harvesting and handling by workers that harbour the pathogens may be a contamination source; thus, cross-contamination is another factor that needs to be considered in spreading antimicrobial-resistant bacteria from foods to humans [67]. In addition, the potential implication of untreated surface water or wastewater for irrigation purposes in Edo State, Nigeria, cannot be overemphasized. An increase in AMR gene carriage in soil has been documented for years [68], pinpointing the role of soil as a reservoir for genes and antibiotic-resistant pathogens. Given that the soil community could harbour these genes and AMR bacteria, contamination during postharvest may also arise from this source in RTE salads and fresh vegetables. This could be an essential route for the spread of antibiotic-resistant pathogens.

Soré et al. [48] documented that resistance to the aminoglycoside, fluoroquinolones and sulfonamides classes was dominant in fresh-cut salads and wastewater samples. An average of ~50% of ESBL-*E*. *coli* isolates from our study were resistant to aminoglycoside and fluoroquinolones, with 35.9% resistant to the sulfonamides class of antimicrobials. Zurfluh et al. [47] reported that all isolates from the investigated fresh vegetables were resistant to cephalothin and ampicillin, while 53 (88.3%) were resistant to cefotaxime. All our isolates were resistant to cefotaxime and ceftazidime, with additional resistance to cefpodoxime 96.9% (62/64), ceftriaxone 98.4% (63/64) and ampicillin 48.4% (31/64). Among 12 antibiotics screened by Ramadan et al. [69], only imipenem seems to have remained resilient, which was in line with the carbapenems activity from our study. The high resistance to fluoroquinolones, penicillin, tetracyclines and aminoglycosides is probably a result of easy access, increased use and non-regulation of these antibiotics on the Nigerian market.

Manure from food animals, usually used as a soil fertilizer, may harbour different resistant bacteria and resistance encoding genes from the gut microbiota of food animals [65]. This phenomenon has become a major problem for public health, not only in underdeveloped countries but also in high-performing, socio-economically developed countries. ESBL *E*. *coli* was identified in vegetables from vendors of market origin in our study and has also been detected in the Netherlands [70] and Tunisia [27]. It is important to note that vegetables that harbour ESBL *E*. *coli* from open markets are usually consumed uncooked or undercooked, where the potential for transfer to humans cannot be ruled out [7]. Outbreaks of foodborne origin (vegetables) consequent of ESBL-*E*. *coli* isolates have been documented [71,72]. Above 50% of ESBL *E*. *coli* recovered could be disseminated via conjugation through ESBL determinants to other bacteria [27]. The horizontal spread of antibiotic-resistant determinants between soil and human bacteria has been reported previously [73]. The transfer of beta-lactamase elements in *E*. *coli* strains could be linked with *IncI1* replicon plasmid acquisition [74].

Our results correlate with other studies indicating vegetables as a possible route for the dynamic diffusion of AMR genes in the community [58]. The *bla*_ESBL_ and other resistance genes on vegetables have been described as existing predominantly in opportunistic and saprophytic bacteria, which are thought to represent a background reservoir of antibiotic resistance genes [22]. Iseppi et al. [75] reported that PCR confirmed the occurrence of *bla*_*CTX*-M-15_ in the ESBL *E*. *coli* studied. Richter et al. [63] Genotypic characterisation showed the widespread prevalence of CTX-M-1, followed by SHV and TEM. CTX-M14 and CTX-M15 are widespread in the environment, food animals and humans in Europe [76,77]. *bla*_TEM-1_ and *bla*_TEM-15_ have also been reported from vegetables in Finland [44]. Similar β-lactamase genes to those from our study have been reported in Southern Thailand [45]. ESBL groups identified from our research (CTX-M-15 and CTX-M-1) also correspond to ESBL groups previously reported in Tunisia [27] from vegetables, irrigation water and soil; and in *E*. *coli* from healthy humans, food-producing animals, and food [74,78]. Hence, contamination by ESBL *E*. *coli* from farm environments and vegetable samples could have occurred from both animal and human origins in Nigeria. The probable consequence of untreated irrigation water can also not be overemphasized as contributing to the spread of MDR pathogens. CTX-M-15 ESBL was detected in our study and has been identified as the most common type of ESBL in Gram-negative bacteria worldwide from different sources, including humans, animals, wildlife and wastewater samples [58].

The finding of resistance genes (*bla*_CTX-M-15_, *bla*_CTX-M-1_, and *bla*_TEM_) commonly linked to human sources [44] highlights the potential transmission route of AMR via food products and emphasizes the importance of hygiene measures. Zurfluh et al. [47] reported that 38.5% (10/26) isolates harboured *bla*_CTX-M-15_, and 3.8% (1) were positive for *bla*_*CTX*-M-1_. This is not in line with the findings from our study, as 10.9% (7) strains harboured *bla*_CTX−M−15_, while 76.6% (49) harboured *bla*_CTX−M−1._ Ramadan et al. [69] reported that 90.4% (179/198) ESBL isolates contained ≥1 *bla*_CTX-M_ gene, which was similar to the 85.9% (55/64) observed for our isolates. The prevalence of *bla*_CTX-M-15_ determinants in isolates has been documented in India following preceding studies from clinical isolates from South India and Delhi [79]. The group CTX-M-15 has progressively been described in community isolates and reported as the utmost Gram-negative ESBL bacteria in Europe [80]. Also, the CTX-M-15 group is especially prevalent in samples from the medical area in Germany [81], while the CTX-M-1 group more often occurs in samples from livestock [82]. The CTX-M-1 group was the most commonly found in this study (49/64; 76.6%, Table 3), which points toward a predominantly livestock origin of the ESBL isolates. Community-onset ESBL-associated infections, including urinary infection and sepsis, are principally caused by *E*. *coli* having *bla*_CTX-M_ ESBLs [54]. Therefore, the relevant β-lactamase genes might contribute to the presence of ESBLs in community-transmitted infections from animal sources to humans via food chains.

The isolates in the present study carried AMR genes conferring resistance against critically essential antimicrobials [83], including aminoglycosides, third- and fourth-generation cephalosporins, tetracyclines and quinolones. All ESBL *E*. *coli* isolates from our study were MDR, and some carried integrons, as this characteristic is typical of ESBL-producing bacteria [84]. However, this was different from a survey by Soré et al. [48], where relatively less (93.23%) of the isolates showed MDR (94.40% in wastewater and 91.66% in salads). Co-expression of β-lactamase genes may result in stronger ESBL production and increased antibiotic resistance or MDR [45]. Antibiotic resistance genes encoding 12 major classes of antibiotics, including β-lactam antibiotics, aminoglycoside, macrolide, fluoroquinolone, and others, were found in vegetable samples from the USA, which were similar to the genes detected from samples from our study [46]. Compared to our findings, class 1 integrons were previously identified in MDR beta-lactamase-positive isolates [63,85]. Class II integron was not identified by Richter et al. [63], while it was identified at a low prevalence of 3.1% (2) in our study. Bezanson et al. [86] recounted that class 2 integrons were primarily identified in Canada, accompanied by class 1 integrons from salad vegetables. This is contrary to the findings from our study, where the class I integron was predominant. The diversity of resistance phenotypes observed among the ESBL-*E*. *coli* isolates showed that the potential spread of resistant bacteria is not the consequence of disseminating a unique strain. It instead appears that genetic variations of ESBLs aren’t narrowly restricted or localized to a singular ecosystem. Farming practices, such as selecting organic fertilizers, types of irrigation water, and sanitation of vegetable processing environments, may affect the composition of the microbiome and antibiotic resistome in vegetables. Understanding the various potential pathways for antibiotic resistome dissemination through cultivation-irrigation-manure microbiomes is critical for mitigating antibiotic resistance. The limitations of this study are the fact that a single state from Nigeria was covered, and state-of-the-art molecular tools such as Whole Genome Sequencing (WGS), multilocus sequence typing (MLST) were not carried out to determine the sequence types (ST), and clones that are circulating with the farms and the market environments. However, the research group is currently working on attracting funding for such aspects of the research to monitor the microbiome, virulome and resistome using metagenomic tools.

## Conclusion

Findings from this study represent the first investigation on the identification and characterization of ESBL *E*. *coli* recovered from farm environments, fresh produce and salads in Edo State, Nigeria. This work showed that fresh vegetables and salads could harbour ESBL-*E*. *coli*, particularly fresh produce from farms that use untreated water for irrigation. Antimicrobial-resistant pathogens can spread to humans via the food chain, especially when these products are consumed uncooked. Suitable measures, for example, improvement of water quality and agricultural practices, need to be implemented, as well as established global regulatory guidelines to ensure public health and consumer safety. Close antimicrobial resistance surveillance in bacteria from a one-health perspective is crucial to source-track and mitigate the development and spread of such bacteria. Findings from this study could serve as baseline data to evaluate the human health risk and the potential transmission associated with the consumption of food vegetables. The subsequent aspect of this study includes expanding the survey to other zones in Nigeria, use of whole genome sequencing and multilocus sequence typing to characterize the isolates further to understand the exact clones that are circulating in the farm environments across Nigeria,

## Supporting information

S1 Table Primers used in this study. (DOCX)
