# Supplementary material for: Multidrug-resistant extended spectrum β-lactamase (ESBL)-producing Escherichia coli from farm produce and agricultural environments in Edo State, Nigeria
Source: PLoS One. 2023 Mar 10;18(3):e0282835. doi: 10.1371/journal.pone.0282835 (PMC10004523; doi:10.1371/journal.pone.0282835)
Supplement: S1 Table — (DOCX) [file pone.0282835.s001.docx]

**PLoS ONE**

Supporting Information file

**Multidrug-resistant extended spectrum β-lactamase (ESBL)-producing *Escherichia coli* from farm produce and agricultural environments in Edo State, Nigeria**

Etinosa O. Igbinosa ^1, ¶^*, Abeni Beshiru ^1,2¶^, Isoken H. Igbinosa^1,3 ¶^, Gyu-Sung Cho^4, ¶^ and Charles M.A.P. Franz ^4, ¶*^

^1^Applied Microbial Processes & Environmental Health Research Group, Faculty of Life Sciences, University of Benin, Private Mail Bag 1154, Benin City 300283, Nigeria

^2^ Department of Microbiology, College of Natural and Applied Sciences, Western Delta University, Oghara, Delta State, Nigeria

^3^ Department of Environmental Management & Toxicology, Faculty of Life Sciences, University of Benin, Private Mail Bag 1154, Benin City 300283, Nigeria

^4^Department of Microbiology and Biotechnology, Max Rubner–Institut, Federal Research Institute of Nutrition and Food, Hermann-Weigmann-Straße 1, Kiel 24103, Germany

^¶^These authors contributed equally to this work

***** Corresponding author E-mail: [Etinosa.Igbinosa@uniben.edu](mailto:Etinosa.Igbinosa@uniben.edu) (EOI)

**Running title**: Multidrug-resistant, ESBL-producing *E. coli* from farm produce and agricultural environments

**S1 Table. Primers used in this study**

| **Target**  **Genes** | **Primer sequences (5’ – 3’)** | **Annealing**  **Temperature** | **Amplicon**  **size (bp)** | **References** |
| --- | --- | --- | --- | --- |
| *bla*_SHV_ | F-AGCCGCTTGAGCAAATTAAAC  R-ATCCCGCAGATAAATCACCAC | 58°C | 713 | Dallenne et al. [1] |
| *bla*_TEM_ | F-CATT TCCG TGTC GCCC TTATTC  R-CGTT CATC CATA GTTG CCTGAC | 58°C | 800 | Dallenne et al. [1] |
| *bla*_CTX−M−15_ | F-CACACGTGGAATTTAGGGACT  R-GCCGTCTAAGGCGATAAACA | 56°C | 996 | Sidjabat et al. [2] |
| *bla*_CTX−M−1_ | F-TTAGGAARTGTGCCGCTGYA  R-CGATATCGTTGGTGGTRCCAT | 60°C | 561 | Dallenne et al. [1] |
| *bla*_CTX−M−2_ | F-ATGA TGAC TCAG AGCA TTCG  R-TGGG TTAC GATT TTCG CCGC | 56°C | 866 | Islam et al. [3] |
| *bla*_CTX−M−8_ | F- AACRCRCAGACGCTCTAC  R- TCGAGCCGGAASGTGTYAT | 60°C | 688 | Islam et al. [3] |
| *bla*_CTX−M−9_ | F- ATGG TGAC AAAG AGAG TGCA  R- CCCT TCGG CGAT GATT CTC | 60°C | 870 | Islam et al. [3] |
| *bla*_OXA−1_ | F-GGCACCAGATTCAACTTCAAG  R-GACCCCAAGTTTCCTGTAAGTG | 58°C | 564 | Dallenne et al. [1] |
| *bla*_OXA−47_ | F-TCAACTTTCAAGATCGCA  R-GTGTGTTTAGAATGGTGA | 50°C | 609 | Islam et al. [3] |
| *bla*_NDM−1_ | F-GGTTTGGCGATCTGGTTTTC  R-CGGAATGGCTCATCACGATC | 57°C | 465 | Islam et al. [3] |
| *bla*_CMY−2_ | F-GACAGCCTCTTTCTCCACA  R-TGGAACGAAGGCTACGTA | 50°C | 1143 | Islam et al. [3] |
| *tet*A | F-GTAA TTCT GAGC ACTG TCGC  R-CTGC CTGG ACAA CATT GCTT | 62°C | 937 | Saenz et al. [4] |
| *tet*B | F-CTCAGTATTCCAAGCCTTTG  R-CTAAGCACTTGTCTCCTGTT | 57°C | 416 | Saenz et al. [4] |
| *tet*M | F-GTGGACAAAGGTACAACGAG  R-CGGTAAAGTTCGTCACACAC | 58°C | 406 | Ng et al. [5] |
| *sul*1 | F-TGGT GACG GTGT TCGG CATTC  R-GCGA GGGT TTCC GAGA AGGTG | 63°C | 789 | Saenz et al. [4] |
| *sul*2 | F-CGGC ATCG TCAA CATA ACC  R-GTGT GCGG ATGA AGT CAG | 50°C | 722 | Saenz et al. [4] |
| *sul*3 | F-CATTC TAGAAAA CAGTCG TAGTTCG  R-CATCTGC AGCTAAC CTAGG GCTTTGGA | 51°C | 990 | Saenz et al. [4] |
| *ant(4´)-Ia* | F-CTGCTAAATCGGTAGAAGC  R-CAGACCAATCAACATGGCACC | 58°C | 172 | Schmitz et al. [6] |
| *aacC*(3)-1 | F-ACCTACTCCCAACATCAGCC  R-ATATAGATCTCACTACGCGC | 60°C | 169 | van de Klundert  and Vliegenthart [7] |
| *qnr*A | F- AGAGGATTTCTCACGCCAGG  R- TGCCAGGCACAGATCTTGAC | 56°C | 580 | Islam et al. [3] |
| *qnr*B | F-GGMATHGAAATTCGCCACTG  R-TTTGCYGYYCGCCAGTCGAA | 56°C | 264 | Islam et al. [3] |
| *qnrC* | F- GGGTTGTACATTTATTGAATC  R- TCCACTTTACGAGGTTCT | 52°C | 307 | Wang et al. [8] |
| *qnr*S | F-GCAAGTTCATTGAACAGGCT  R-TCTAAACCGTCGAGTTCGGCG | 60°C | 428 | Farajzadeh-Sheikh et al. [9] |
| *cat::p*C194 | F-CAATCCAAGGAATCATTGAAATCGG  R-AAAGCCAGTCATTAGGCCTATCTG | 58°C | 472 | Argudın et al. [10] |
| *cat::p*C221 | F-TGGA AGTT GTAA ATAA AAAT AAA GTG  R-CAAT CCAA GGAA TCAT TGAA ATCGG | 59°C | 269 | Argudın et al. [10] |
| *cat::p*C223 | F-AGGA TATG AACT GTAT CCTG CTTTG  R-AATA ATGA AACA TGGT AACC ATCAC | 59°C | 464 | Argudın et al. [10] |
| *int*I1 | F-GGTCAAGGATCTGGATTTCG  R-ACATGCGTGTAAATCATCGTC | 60°C | 436 | Rizk and El-Mahdy [11] |
| *int*I2 | F-CACGGATATGCGACAAAAAGG  R-TGTAGCAAACGAGTGACGAAATG | 60°C | 788 | Rizk and El-Mahdy [11] |

**References**

1. Dallenne C, Da Costa A, Decre D, Favier C, Arlet G. Development of a set of multiplex PCR assays for the detection of genes encoding important β-lactamases in Enterobacteriaceae. J Antimicrob Chemother. 2010; 65: 490–495.
2. Sidjabat HE, Paterson DL, Adams-Haduch JM, Ewan L, Pasculle AW, Muto CA, et al. Molecular epidemiology of CTX-M-producing *Escherichia coli* isolates at a tertiary medical center in western Pennsylvania. Antimicrob Agents Chemother. 2009; 53:4733–4739.
3. Islam MA, Talukdar PK, Hoque A, Huq M, Nabi A. Emergence of multidrug-resistant NDM-1-producing Gram-negative bacteria in Bangladesh. Eur J Clin Microbiol Infect Dis. 2012; 31: 2593–600.
4. Saenz Y, Brinas L, Dominguez E, Ruiz J, Zarazaga M, Vila J, et al. Mechanisms of resistance in multiple-antibiotic-resistant *Escherichia coli* strains of human, animal, and food origins. Antimicrob Agents Chemother. 2004; 48(10):3996–4001.
5. Ng LK, Martin I, Alfa M, Mulvey M. Multiplex PCR for the detection of tetracycline resistant genes. Mol Cell Probes. 2001; 15:209–215.
6. Schmitz FJ, Fluit AC, Gondolf M, Beyrau R, Lindenlauf E, Verhoef J, et al. The prevalence of aminoglycoside resistance and corresponding resistance genes in clinical isolates of staphylococci from 19 European hospitals. J Antimicrob Chemother. 1999; 43:253–259.
7. van de Klundert JAM, Vliegenthart JS. PCR detection of genes coding for aminoglycoside-modifying enzymes. In: D. H. Persing, T. F. Smith, F. C. Tenover, and T. J. White (ed.), Diagnostic molecular microbiology. American Society for Microbiology, Washington, D.C. 1993; p. 547–552.
8. Wang M, Guo Q, Xu X, Wang X, Ye X. New plasmid-mediated quinolone resistance gene, *qnrC*, found in a clinical isolate of *Proteus mirabilis*. Antimicrob Agents Chemother. 2009; 53: 1892–1897.
9. Farajzadeh-Sheikh A, Veisi H, Shahin M, Getso M, Farahani A. Frequency of quinolone resistance genes among extended-spectrum β-lactamase (ESBL)-producing *Escherichia coli* strains isolated from urinary tract infections. Trop Med Health. 2019; 47(1): 19.
10. Argudın MA, Tenhagen BA, Fetsch A, Sachsenrder J, Kasbohrer A, Schroeter A, et al. Virulence and resistance determinants of German *Staphylococcus aureus* ST398 isolates from nonhuman sources. Appl Environ Microbiol. 2011; 77:3052–3060.
11. Rizk DE, El-Mahdy AM. Emergence of class 1 to 3 integrons among members of Enterobacteriaceae in Egypt. Microb Pathog. 2017; 112: 50–56.
